# Facile synthesis and biological evaluation of glomuferrin and rhizoferrin as ferroptosis inhibitors in rice blast disease

**DOI:** 10.1007/s13659-026-00619-x

**Published:** 2026-05-04

**Authors:** Anna Fusetti, Francesca Annunziata, Michael S. Christodoulou, Andrea Pinto, Andrea Kunova, Salvatore Princiotto, Sabrina Dallavalle

**Affiliations:** Department of Food, Environmental and Nutritional Sciences, University of Milan, via Celoria 2, 20133 Milan, Italy

**Keywords:** Ferroptosis, Rice blast disease, Siderophore, Iron, Chelation, Appressorium

## Abstract

Iron homeostasis has recently emerged as a key determinant in the early stages of plant–pathogen interactions, particularly in phytopathogens that exploit iron-dependent cell death (known as ferroptosis) to initiate host invasion. In *Pyricularia oryzae*, the causal agent of rice blast disease, appressorium formation is tightly linked to ferroptotic events in germinating conidia. Accumulation of intracellular Fe^3^⁺ and reactive oxygen species (ROS) promotes lipid peroxidation, ultimately triggering conidial cell death and, consequently, appressorium maturation. Therefore, inhibition of ferroptosis by targeting fungal iron homeostasis represents a promising strategy to suppress appressorium development and block subsequent host infection. Rhizoferrin, an α-hydroxy carboxylate siderophore secreted by *Rhizopus microspora*, was identified as a suitable precursor of its ring-closed derivative glomuferrin. The obtainment of an unexpected succinimide intermediate within the synthetic pathway enabled the access to both rhizoferrin and, to our knowledge for the first time, glomuferrin. Chelation evaluation revealed that rhizoferrin exhibits significantly higher affinity for iron than glomuferrin. The effects of these siderophores on conidial germination and appressorium formation were evaluated in wild-type and strobilurin-resistant *P. oryzae* strains, at concentrations ranging from 5 mM to 200 μM. The promising activity observed, especially for rhizoferrin, highlights the potential of α-hydroxy carboxylate siderophores as ferroptosis inhibitors and offers new perspectives for their development in crop protection.

**Graphical Abstract:**

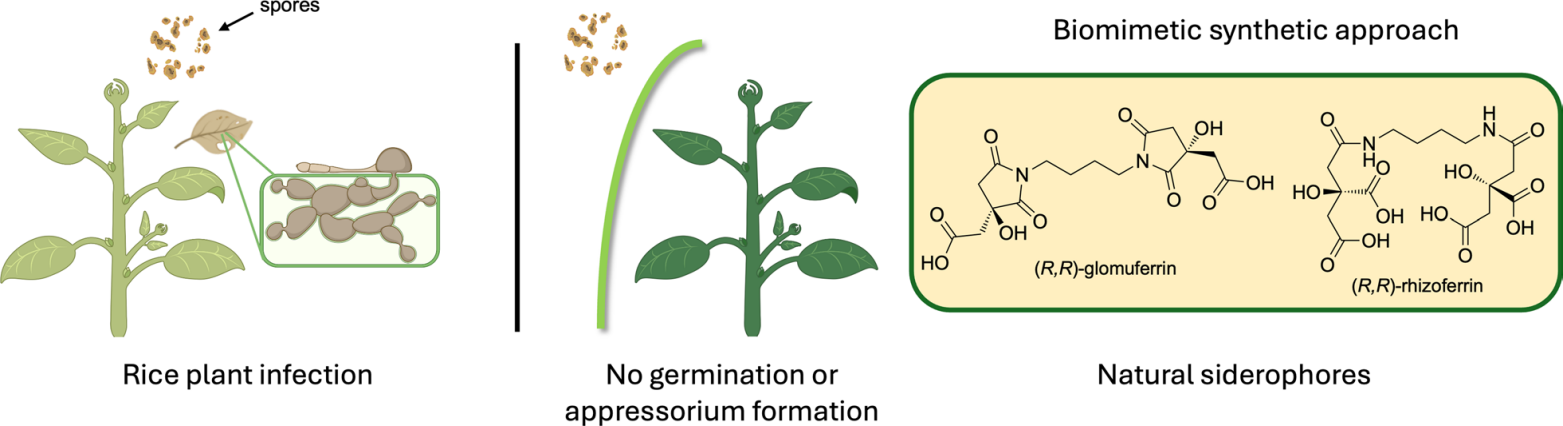

**Supplementary Information:**

The online version contains supplementary material available at 10.1007/s13659-026-00619-x.

## Introduction

Iron is an essential micronutrient playing a central role in a wide range of cellular processes, including respiration, photosynthesis, and DNA synthesis [1]. In its ferric form (Fe^3^⁺), iron is employed as a critical cofactor in numerous redox enzymes and metalloproteins. However, despite its abundance, Fe^3^⁺ is largely insoluble under physiological conditions, presenting a major challenge for biological acquisition [2]. To overcome this limitation, plants and microorganisms have evolved highly specialized strategies for iron uptake, mobilization, and storage. In plants, Fe^3^⁺ is reduced to the more soluble ferrous form (Fe^2^⁺) at the root surface or chelated for transport by specialized structures acting as chelating agents, known as phytosiderophores [3, 4]. Microorganisms, such as bacteria and fungi, produce low-molecular-weight siderophores that bind Fe^3^⁺ with high affinity, facilitating its solubilization and uptake through specific transport systems.

Over the past decade, there has been growing interest in the iron-mediated mechanisms of plant infections caused by phytopathogenic microorganisms. A notable example is rice blast disease, caused by the fungus *Pyricularia oryzae,* which has been recently reported to infect rice crops through a regulated cell death process known as ferroptosis [5]. The infection process starts with a specialized structure, called appressorium, which is formed after the conidium germination. The maturation of the appressorium is strictly dependent on the ferroptotic events taking place in the conidium, coupled with the accumulation of high levels of endogenous reactive oxygen species (ROS). This process is finely regulated by intracellular iron levels, which induce lipid peroxidation through the activity of NADPH oxidases (NOXs), ultimately leading to cell death [6, 7]. During the appressorium maturation, melanization of its cell wall allows to generate high inner pressure due to solute accumulation, thus providing the extrusion of a penetration peg ready to breach the plant leaf epidermis and differentiate into invasive hyphae [1, 6]. For this reason, the use of iron-coordinating compounds could be an efficient strategy to subtract Fe^3+^ from phytopathogens, thus inhibiting ferroptosis and suppressing their infectivity.

**Fig. 1:**
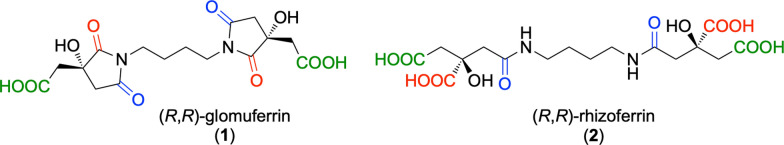
Chemical structure of (*R*,*R*)-glomuferrin and its hydrated product (*R*,*R*)-rhizoferrin

Naturally occurring glomuferrin is a hydroxy-polycarboxylate secreted by arbuscular mycorrhizal (AM) fungi to promote plant growth [8]. To date, little is known about its physico-chemical and biological properties. Structural elucidation by Winkleman and coworkers [8] led to the identification of a succinimide fragment, containing a β-hydroxy acid which is thought to arise from dehydration of a distinctive siderophore, known as rhizoferrin, commonly secreted by some microorganisms (Fig. 1).

(*R*,*R*)-Rhizoferrin was firstly isolated from a fungal pathogen *Rhizopus microsporus*, responsible for mucormycosis in dialysis patients, and typically produced by different Mucoromycota and Entomophthoromycota (previously Zygomycota) [9]. Noteworthy, fungal phytopathogens generally produce hydroxamate- or catecholate-type siderophores, making polycarboxylates rhizoferrin (and consequently glomuferrin) atypical substrates for the microorganism uptake system. Consequently, the presence of such siderophores would allow iron chelation without necessarily guaranteeing the transport of the resulting complex within the fungal cell. On the other hand, rhizoferrin enantiomeric form (*S*,*S*) is produced by an opportunistic human pathogenic bacteria responsible for nosocomial infections, *Ralstonia* (formerly *Burkholderia* or *Pseudomonas*) *pickettii*, grown under iron-limited conditions. [10] The iron-chelating ability of both the enantiomeric forms of this siderophore is reflected in their high affinity constant log K_ml_ = 25.3 [11]. This is largely due to the formation of a hexacoordinated complex, which adopts a cage-like structure with octahedral geometry, enabling highly efficient Fe^3+^ chelation [12, 13].

Given the recognition of hydroxamates by *P.* *oryzae* transport proteins, [14] attention was directed toward the hydroxy carboxylates glomuferrin and rhizoferrin, which are unlikely to be recognized by the pathogen’s uptake systems. Noteworthy, although glomuferrin has been described as a natural siderophore, solely its plant growth-promoting activity has been reported so far. Hence, we aimed to investigate its iron-chelating potential for possible application in the treatment of fungal infections in crop protection. To this aim, we undertook the design and synthesis of the glomuferrin scaffold. As no synthetic approaches have been reported to date, our objective was to develop a viable synthetic route to obtain sufficient amount of the compound for biological evaluation. Since iron-mediated ferroptosis has been identified indispensable for *P.* *oryzae* infection, [5, 15, 16] the ability of glomuferrin to inhibit *P.* *oryzae* spore germination and appressorium formation was also assessed.

## Results and discussion

### Chemistry

**Scheme 1.**
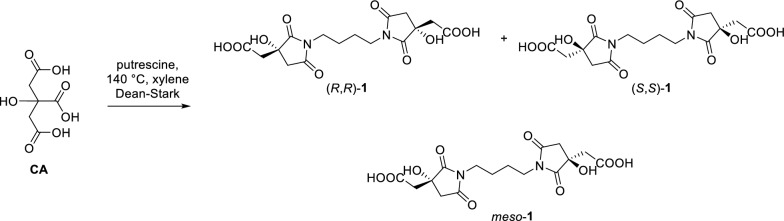
Reaction of citric acid (**CA**) and putrescine to give (*R,R*)-**1**, (*S,S*)-**1** and *meso*-**1**

Initially, the synthesis of glomuferrin was approached following methods previously described for the preparation of succinimide-based polymers, neat or in xylene [17]. The reaction was carried out in xylene to enhance the solubility of the starting materials, facilitating the amide bond formation followed by the dehydration step. 1,4-Diaminobutane (commonly known also as putrescine) was used as the nitrogen donor moiety, in stoichiometric ratio 1:2 with respect to citric acid (**CA**). No trace of glomuferrin was detected using the regular condenser, or adding molecular sieves to the reaction environment. On the other hand, the use of Dean-Stark apparatus was essential for a suitable removal of water, resulting in complete consumption of **CA** and the formation of the succinimide moiety (Scheme 1).

Specifically, condensation of achiral **CA** with putrescine was followed by ring closure and delivered the glomuferrin skeleton, containing two new stereogenic centers. Since no stereoselection took place under the reported conditions, compound **1** was isolated as racemic mixture of (*R*,*R*) and (*S*,*S*) enantiomers, along with the *meso* diastereoisomer. Purification by flash chromatography afforded *rac-1* (22%) and *meso-1* (28%), as confirmed by mass spectrometry (molecular peak at 399.10, corresponding to the [M–1]^–^) and HPLC analysis on chiral stationary phase (Fig. S1).

With optically inactive glomuferrin in hand, we planned a stereoselective synthetic strategy to prepare the naturally occurring enantiomeric form (*R*,*R*)-**1**. To this end, we envisioned using rhizoferrin as a suitable glomuferrin precursor [8].

**Scheme 2.**
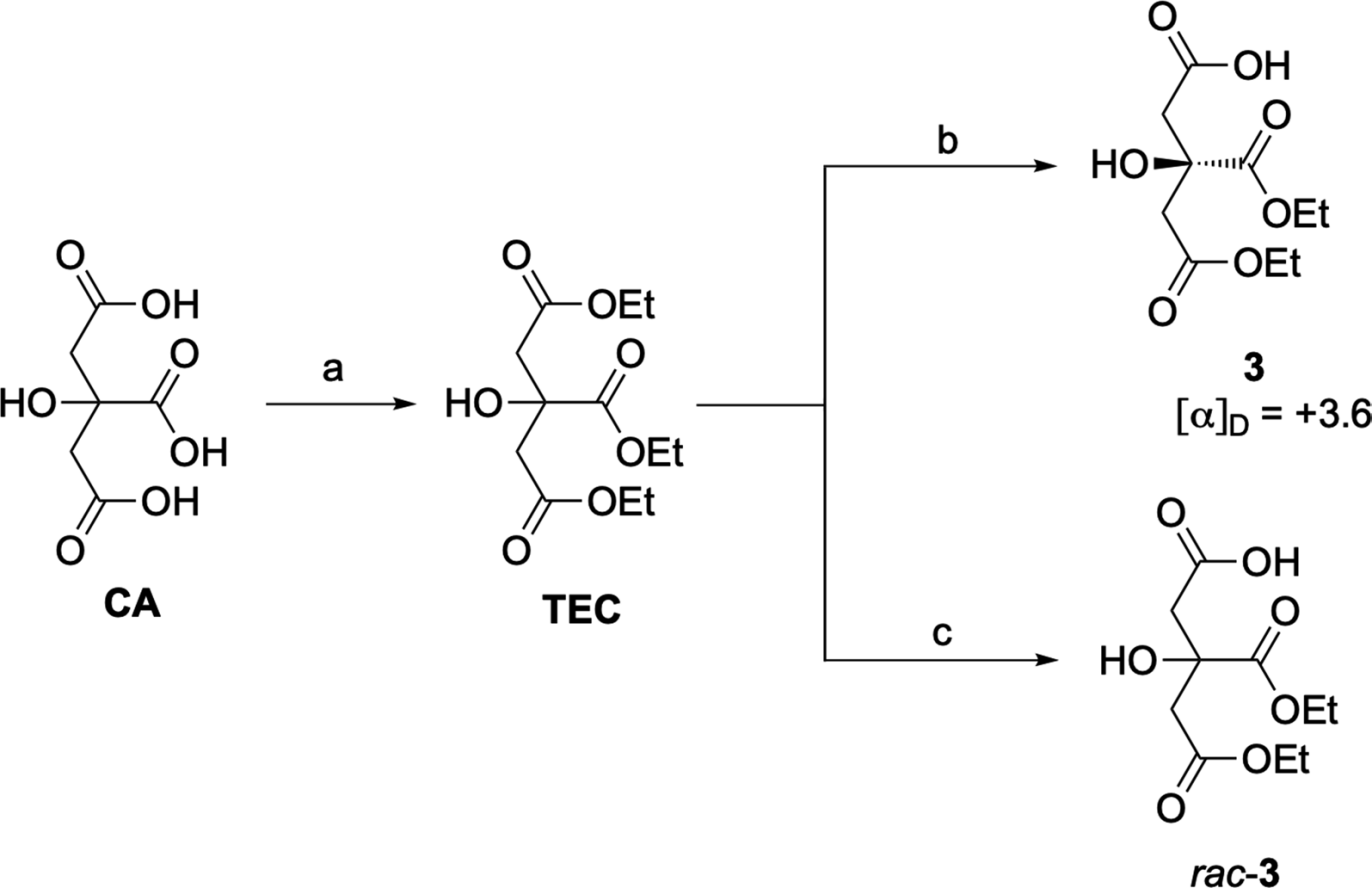
Preparation of enantiopure monoacid **3** and *rac*-**3** starting from citric acid **CA**. Reagents and conditions: **a** SOCl_2_, EtOH, 78 °C, 16 h, 94%; **b** *imm*-CaLB, phosphate buffer pH = 7.4, 40 °C, 60 h, 93%; **c** EtONa/EtOH

To date, several approaches for the synthesis of (*R*,*R*)-rhizoferrin **2** have been reported [9, 18]. Interestingly, Serbian and colleagues performed the desymmetrization of achiral triethyl citrate (**TEC**) by treatment with immobilized lipase B from *Candida* *antarctica* (*imm*-CaLB) in phosphate buffer (pH = 7.4), selectively hydrolyzing only one of the two terminal esters [18]. Following this protocol, **CA** was converted to **TEC**, easily hydrolysed to monoacid **3** in a regio- and stereoselective manner, as confirmed by the observed [α]_D_ =  + 3.6, consistent with values previously reported in literature (Scheme 2) [9, 19]. To further confirm the enantioselective conversion of **TEC** to **3** by the biocatalyzed approach, HPLC analysis on chiral stationary phase (Kromasyl AmyCoat 5 (4.6 × 250 mm, 5 μM particle size) was carried out. To this aim, *rac*-**3** was prepared by treatment with EtONa in EtOH and compared with **3**, obtained by CaLB-catalysed hydrolysis (Scheme 2).

**Scheme 3.**
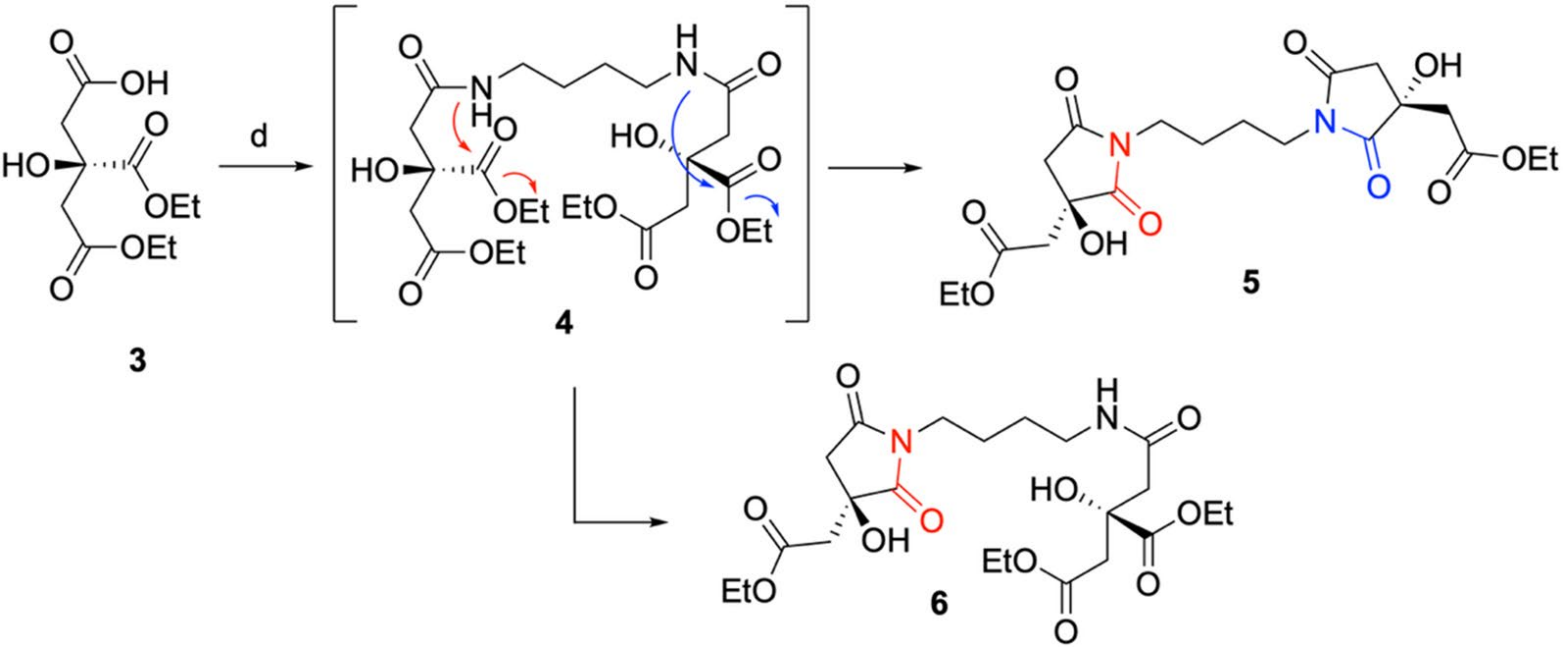
Schematic representation for the obtainment of compounds **5** and **6**. Reagents and conditions: **d** TBTU, HOBt, DIPEA, in DMF, putrescine, 0 °C → rt, 16 h–72 h. **5**: 69%; **6**: traces

Gratifyingly, the elution of a major peak at 11.2 min, present in a 95:5 ratio with its enantiomer (Rt = 12.8 min), confirmed the obtainment of the desired (*R*)-**3** with an approximately 90% *ee* through lipase-based desymmetrization of TEC (Figure S2) [19]. The obtainment of the enantioenriched monoacid **3**, featuring a quaternary stereogenic center unable to further undergo racemization, was essential to proceed with the synthetic pathway toward rhizoferrin and glomuferrin. As the following step, the formation of the amide bond between the carboxylic acid of compound **3** and the amino groups of putrescine proved challenging. Numerous attempts carried out following the reported synthetic protocol [18] consistently resulted in complex crude mixtures that were difficult to be efficiently purified, thus preventing the isolation of the desired dimeric product **4**. In-depth spectroscopic analysis of the collected fractions revealed the presence of a major product characterized by one ethyl ester system (instead of two) and a resonance of the *N*-methylene protons at 3.55 ppm, rather than the expected signal at 3.1–3.2 ppm [18]. Such notable downfield shift, characteristic of the succinimide ring system [20] and previously observed in glomuferrin isolated as *rac*-**1** and *meso*-**1**, indicated the formation of glomuferrin diethyl ester **5** (Scheme 3). Notably, one minor product, consistently present in all isolated fractions, was further purified and identified as the partially ring-closed compound **6**, previously described as imidorhizoferrin [20]. Only traces of rhizoferrin tetraethyl ester **4** were observed, likely indicating its role as intermediate ready to undergo ring closure and formation of **5** and **6**.

Although surprising, these results provided an opportunity to exploit the formation of the cyclic systems occurring in the main product **5**. It is well documented that 5-member amido ester systems can undergo cyclization under alkaline conditions, particularly in the presence of strong excess of base (i.e., 11 eq. of DIPEA [18], as described in the protocol) [21–25].

However, even when the amount of base was reduced (to 5 or 7 equivalents) and the reaction time shortened to 8 h, the main product was never the linear rhizoferrin derivative **4**, but instead compounds **5** and **6**. The obtained result could be easily explained by the formation of the 5-member ring succinimide, possibly favoured by the α-hydroxy group through an intramolecular hydrogen bond with the vicinal ethyl ester.

**Fig. 2:**
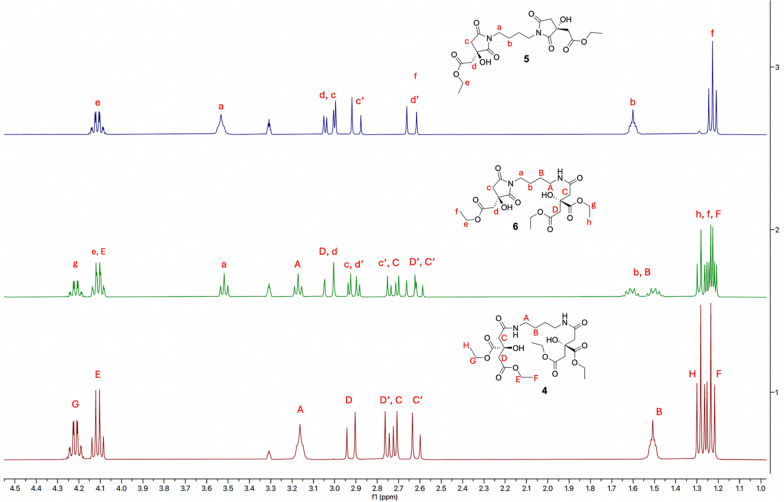
Chemical shift comparison between obtained pure glomuferrin diethyl ester **5**, imidorhizoferrin triethyl ester **6** and rhizoferrin tetraethyl ester **4** (^1^H-NMR in CD_3_OD)

After 16 h, the condensation between **3** and putrescine afforded a crude mixture easily purified by chromatography, giving **5** in 69% yield. Similar results were observed even after 72 h, in sharp contrast with the previously reported literature data [18]. Figure 2 reports ^1^H-NMR spectra of the pure **4**, **5** and **6**, showing marked differences in chemical shifts of diagnostic signals *a* and *A* (α position with respect to the succinimide/amide nitrogen), *e* and *E*/*g* and *G* (CH_2_ protons of the ethyl fragment).

Following confirmation of the reproducible obtainment of glomuferrin diethyl ester **5**, a divergent synthetic approach to stereoselectively obtain both rhizoferrin and glomuferrin from a common intermediate was explored.

**Scheme 4.**
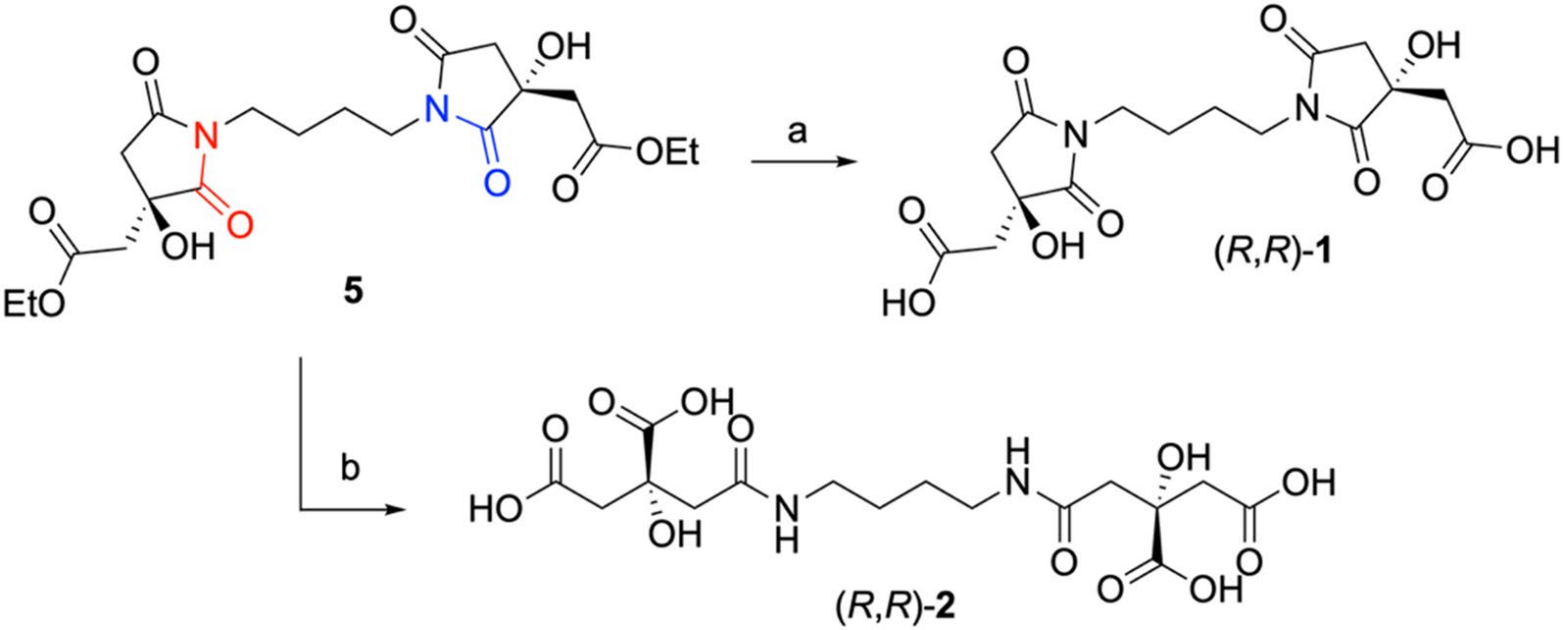
Stereodivergent synthesis of glomuferrin (*R*,*R*)-**1** and rhizoferrin (*R*,*R*)-**2** starting from the common intermediate **5**. Reaction and conditions: **a** i. *imm*-CaLB, distilled water, rt, 16 h; ii. Amberlyst 15^®^, 99%; **b** i. LiOH, in THF/MeOH/H_2_O, rt, 48 h; ii. Amberlyst 15^®^, 99%

Given the susceptibility of the succinimide ring to alkaline hydrolysis (e.g., barium or lithium hydroxide, followed by acidification), a chemoselective hydrolysis to obtain (*R*,*R*)-glomuferrin was investigated. For this purpose, *imm*-CaLB (50 mg/mL) was used as biocatalyst and added to a fine suspension of **5** in distilled water. The complete conversion of the ester moieties to the corresponding carboxylic acids allowed the formation of pure glomuferrin (*R*,*R*)-**1** in quantitative yield (Scheme 4).

On the other hand, the hydrolysis of the diethyl ester intermediate **5** was carried out in presence of LiOH in H_2_O/THF/MeOH, resulting in the concurrent 5-member ring opening. Neutralization of the excess of LiOH and protonation of carboxylates were finally achieved by passing the crude mixture through a small column packed with a cation-exchange sulfonic resin (Amberlyst 15^®^) (Scheme 4). After lyophilization, the natural compound (*R*,*R*)-rhizoferrin (**2**) was obtained as a single product, as confirmed by NMR analysis (multiplet peak at 3.1 ppm), indicating that the succinimide moiety was selectively cleaved at C(2).

### Chelation assay

**Fig. 3:**
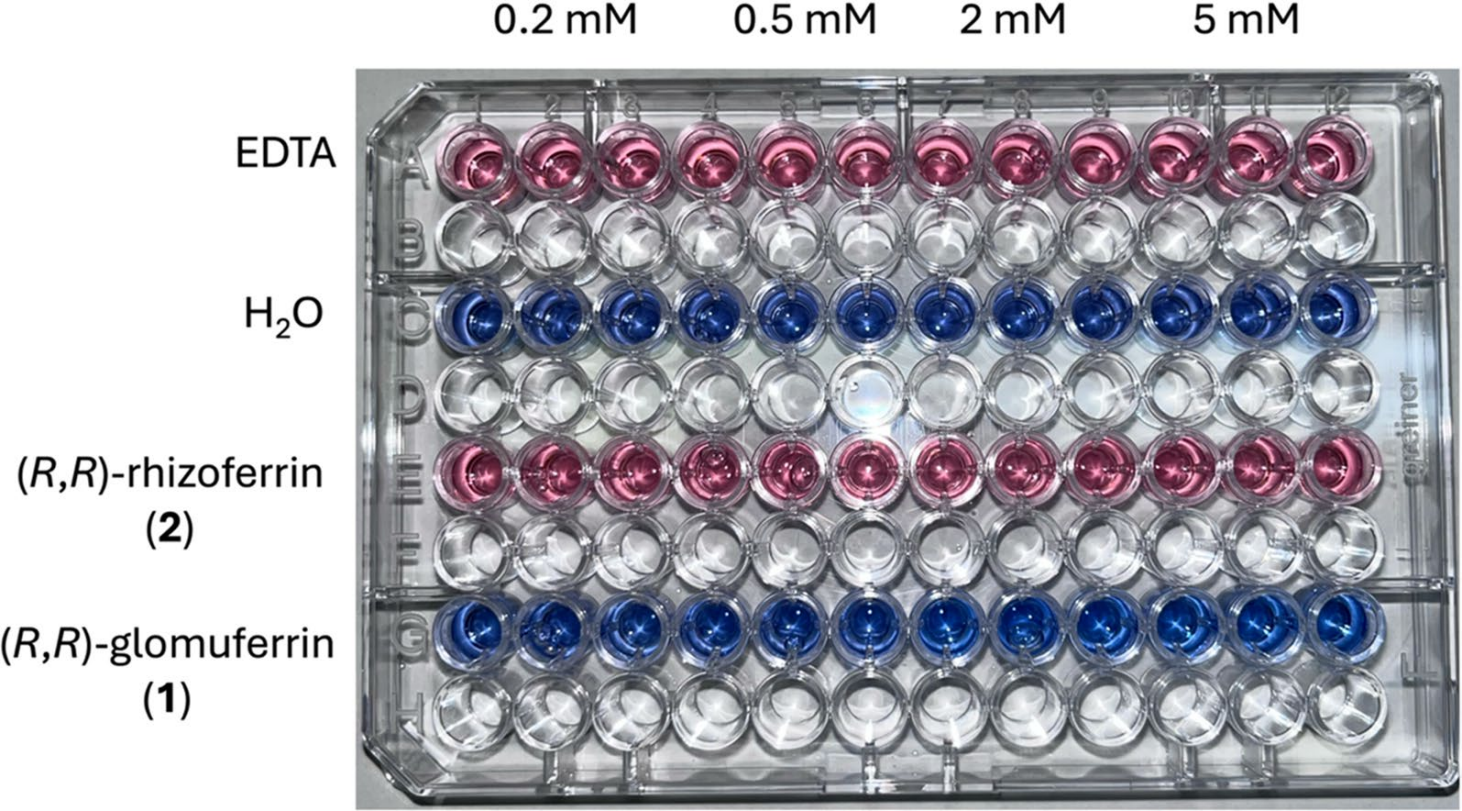
CAS assay performed on EDTA (positive control), H_2_O (negative control), compounds **1** and **2** at 0.2, 0.5, 2 and 5 mM concentrations

The potential of the synthesized compounds to chelate Fe^3+^ was evaluated by colorimetric Chromeazurol S (CAS) assay [26]. To this aim, samples of (*R*,*R*)-glomuferrin **1** and (*R*,*R*)-rhizoferrin **2** were dissolved in H_2_O, treated with the reagent solution and tested in triplicate in a 96-well plate at four different concentrations: 0.2, 0.5, 2 and 5 mM. EDTA was used as positive control, turning the well from blue to pink, while the solvent mixture was considered as negative control, maintaining the blue color of the original solution (Fig. 3).

The well-established ability of rhizoferrin **2** to act as a siderophore [9] was confirmed by the intense pink color similar to the one observed for EDTA. Such chromic shift clearly indicated an iron-chelating potential comparable with the positive control. Surprisingly, once treated with CAS shuttle solution, glomuferrin **1** did not cause any change in color at any of the tested concentrations. This evidence is partially in contrast with other reports describing glomuferrin as a siderophore [8, 20]. In particular, it has never been demonstrated whether glomuferrin is produced by arbuscular mycorrhizal fungi or by dehydration of rhizoferrin occurring during extraction processes [8]. Such a difference in behavior clearly underscores the importance of the α-hydroxy acid moieties of rhizoferrin in coordinating Fe^3^⁺, ultimately enabling the formation of the hexacoordinated 1:1 complex [11].

### Biological activity evaluation

**Fig. 4:**
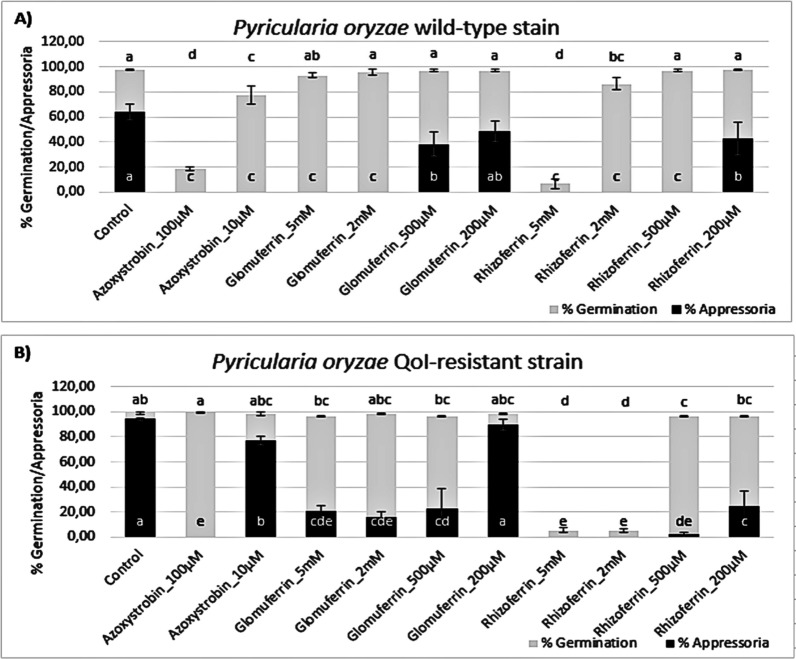
Percent of germination (grey) and appressorium formation (black) of *Pyricularia oryzae* wild-type (*wt*; panel A) and QoI-resistant strain (*res*; panel B) upon treatment with glomuferrin and rhizoferrin at 5, 2, 0.5 and 0.2 mM. Control was water, while azoxystrobin (100 and 10 μM) was used as a standard. Error bars represent the standard deviation. The different letters indicate statistically significant differences among means (*p* > 0.05; for germination above the graph, for appressoria at the base of the graph) calculated by the Tukey post hoc test

The ability of the obtained compounds to inhibit the appressorium formation was tested on spores from two strains of *P. oryzae*: PO21_05 (wild-type, *wt*) and PO21_07 (strobilurin resistant, *res*). Glomuferrin **1** and rhizoferrin **2** were assayed at the same concentrations reported in paragraph 2.2 (chelation assay), to verify any possible correlation between the iron-chelating ability and the biological activity. The results are shown in Fig. 4. In control conditions, both strains showed germination > 95% (grey bar); however, *P.* *oryzae res* displayed much better appressorium formation (94% compared to 64% of the *wt* strain; black bar). Azoxystrobin (QoI fungicide used for *P.* *oryzae* management) was used as a standard. In the *wt* strain (Fig. 4A), the positive control almost completely inhibited germination at 100 µM and significantly reduced it even at 10 µM. In *res* (Fig. 4B) appressorium formation was suppressed at the concentration of 100 µM, while it was almost completely restored (77% compared to 94% in control) at 10 µM. Glomuferrin did not affect the germination of the two strains; however, it strongly inhibited appressorium formation of both strains up to the concentration of 500 µM. On the other hand, rhizoferrin showed much stronger activity, completely inhibiting appressorium formation at 200 µM concentration in both strains. Additionally, it was able to inhibit spore germination at 2 mM concentration.

**Fig. 5:**
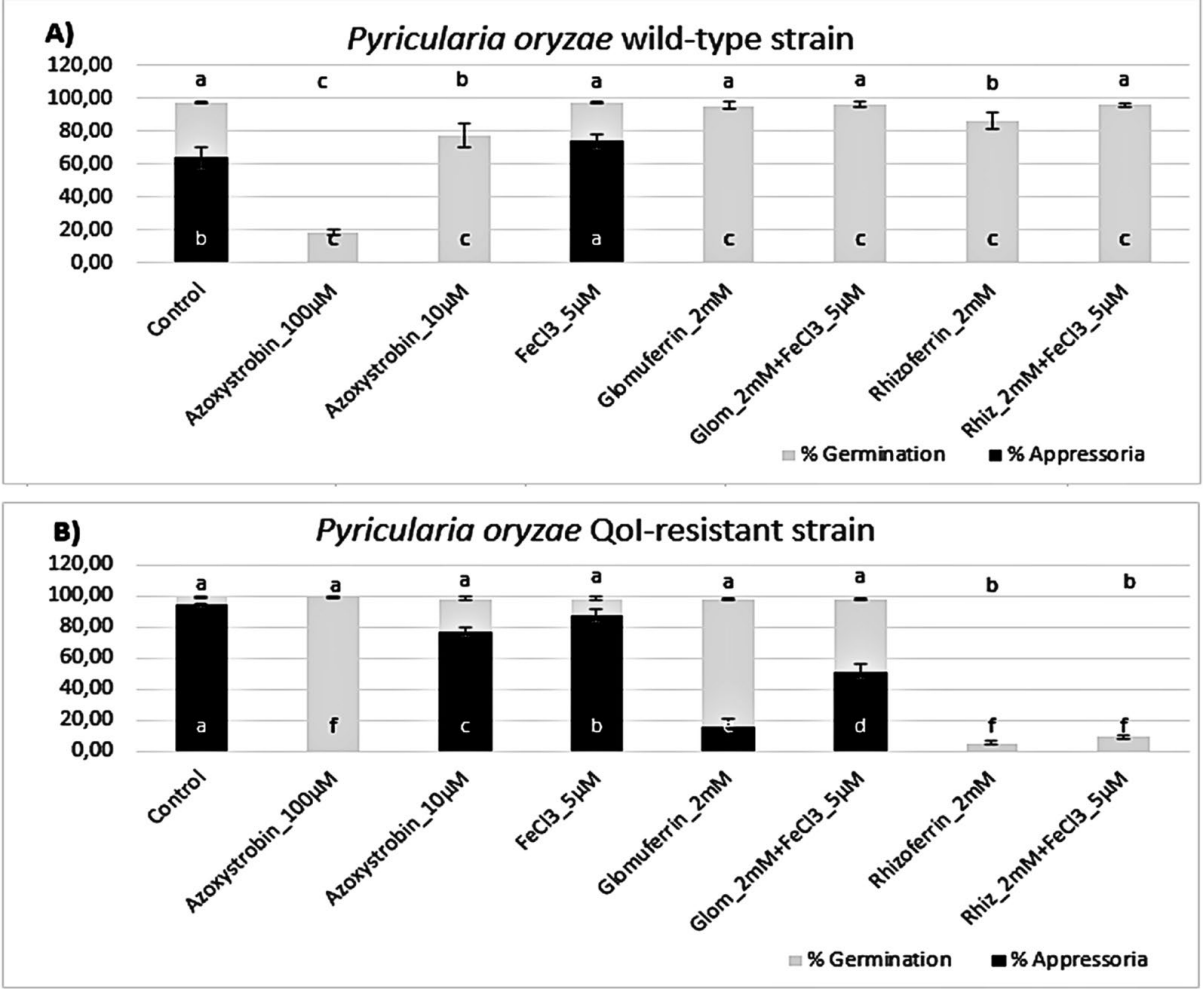
Activity of glomuferrin and rhizoferrin at 2 mM on the germination (grey) and appressorium formation (black) of *Pyricularia oryzae* wild-type (*wt*; panel **A**) and QoI-resistant strain (*res*; panel **B**) in the presence of 5 µM FeCl_3_. Control was water, while azoxystrobin (100 and 10 μM) was used as a standard. Error bars represent the standard deviation. The different letters indicate statistically significant differences among means (*p* > 0.05; for germination above the graph, for appressoria at the base of the graph) calculated by the Tukey post hoc test

To verify that the observed activity is linked to ferroptosis and iron homeostasis, recovery experiments were performed by adding 5 µM Fe^3+^ (Fig. 5). The addition of FeCl_3_ at 5 µM concentration alone improved the formation of appressoria in *wt* (Fig. 5A), while slightly reduced appressoria in *res*-strain (Fig. 5B). When added together with glomuferrin at 2 mM, in the *res*-strain addition of Fe^3+^ partially improved appressorium formation (51%) compared with glomuferrin alone (15%), while no effect was observed in *wt*. In the case of rhizoferrin, addition of Fe^3+^ improved germination of the *wt*-strain, but no appressoria were observed.

Overall, these results support a concentration-dependent inhibition of the germination and appressorium development by both glomuferrin and rhizoferrin. Given the critical role of Fe^3+^ in this process, it is likely that appressorium development is suppressed due to iron unavailability to the fungal pathogen. Generally, glomuferrin showed lower activity compared to rhizoferrin. These results, together with its previously reported ability to promote plant growth, [8] could provide a boost towards *in planta* studies, potentially demonstrating plant protection by two distinct mechanisms of action. Notably, in agreement with the colorimetric assay, rhizoferrin consistently exhibited greater potency than glomuferrin, even completely inhibiting spore germination at the highest tested concentrations. This enhanced activity could be explained by the presence of a more efficiently-coordinating tetracarboxylic moiety in rhizoferrin, compared to the more rigid and less functionalized structure of glomuferrin.

## Conclusion

A novel divergent synthetic pathway for the stereoselective preparation of polycarboxylate siderophores (*R*,*R*)-glomuferrin **1** and (*R*,*R*)-rhizoferrin **2** was designed and successfully carried out. It is worth noting that this is the first total synthesis reported for glomuferrin, approached by a divergent pathway starting from monoacid (*R*)-**3**, obtained via a stereoselective biocatalyzed hydrolysis on **TEC** in presence *imm*-CaLB lipase. Such desymmetrization guaranteed the stability of the quaternary stereocenter against racemization, giving access to enantioenriched (*R*,*R*)-glomuferrin and (*R*,*R*)-rhizoferrin. The solid data supporting the stereoselectivity of the enzymatic hydrolysis, together with the observed ring-closure of such systems, could be useful in the development of new more accessible approaches towards the (*S*,*S*)-enantiomers and optimized analogues. (*R*,*R*)-Rhizoferrin showed good chelating activity in CAS test, and, more importantly, completely inhibited appressoria formation in *P.* *oryzae wt*-strain at 500 μM. Considering the *res*-strain, also spore germination was inhibited at 5 and 2 mM. In contrast, glomuferrin exhibited only weak activity in the CAS assay, which corresponded to a notably lower inhibition of appressorium formation in *P.* *oryzae* compared to rhizoferrin. Overall, these data highlight the critical role of the α-hydroxy acid moiety in the formation of high-affinity iron complexes, a feature that could guide the design of novel potential siderophores acting as ferroptosis inhibitors.

## Experimental section

### Material and methods

All reagents and solvents were purchased from commercial suppliers and used without further purification. *Imm*-CalB lipase (lot n. 316694, 295919, 270027) was purchased by Merck (Milan, Italy) All reactions requiring anhydrous conditions were performed under a positive nitrogen flow (after vacuuming the flask), and all glassware was oven dried. Thin layer chromatography (TLC) analyses were performed using commercial silica gel 60 F_254_ aluminum sheets. Spots were revealed under a UV lamp (λ = 254 or 365 nm). Ninhydrin, phosphomolybdic acid, bromocresol green and Pancaldi solution-based TLC stains were used occasionally as needed. Isolation and purification of the products were performed by flash column chromatography on silica gel 60 (230–400 mesh). Optical rotations were measured using a Jasco *p*-2000 polarimeter. The NMR spectra were acquired using a Bruker NMR Avance NEO 400 MHz spectrometer (^1^H 400 MHz,^13^C 100 MHz). Tetramethylsilane (TMS) was used as an internal standard, and chemical shifts (* δ*) are expressed in ppm. The coupling constants (*J*) are reported in Hz. For the NMR analysis we used CDCl_3_, CD_3_OD, D_2_O, DMSO-*d6* as deuterated solvents. All the spectra were recorded at room temperature (298.15 K). ^1^H NMR signals are indicated with the following abbreviations: s (singlet), d (doublet), t (triplet), q (quartet), dd (doublet of doublets), m (multiplet). HPLC analyses were performed using a JASCO PU-980 Binary HPLC Pump, equipped with a JASCO UV-975 UV–vis detector and a JASCO LC-NetII/ADC interface box. Chiral column employed was a Kromasil 5-AmyCoat (4.6*250 mm). Injection volume: 20 μL. Flow rate: 1.0 mL min^−1^, *n-*hex: *i*PrOH 9:1 + 0.1% TFA (**3** vs *rac*-**3**) or *n*-hex: *i*PrOH 1:9 + 0.2% formic acid. Mass spectrometry analyses were performed at the Mass Spectrometry facility of the Unitech COSPECT at the University of Milan (Italy).

### Chromeazurol S (CAS) assay

*CAS Reagent.* 100 mL graduated glass cylinder was previously washed with 6 M HCl. A solution of hexadecyltrimethylammonium bromide (HDTMA) (22 mg in 6 mL distilled water, 10 mM) was poured into the cylinder and diluted to 40 mL with distilled water. 1.5 mL of ferric iron solution (2.7 mg of FeCl_3_·6 H_2_O in 10 mL 1 M HCl, 1 mM) was added while stirring. Finally, 7.5 mL of Chrome Azurol S (CAS) solution (9.1 mg in 7.5 mL distilled water, 2 mM) was slowly added to the mixture.

In a round bottom flask, a solution of piperazine (4.31 g in 20 mL distilled water, 2.5 M) was cooled to 0 °C and 6.25 mL of concentrated HCl was carefully added. The piperazine solution was added to the CAS/Fe^3+^ solution in the cylinder and then brought to a final volume of 100 mL with distilled water. Lastly, sulfosalicylic acid (102 mg) was added and dissolved to afford a blue solution, stored in a polyethylene falcon in a cool and dark place.

*Chelation Assay.* To prepare the stock solutions of the tested compounds, 1 mg was dissolved in 1 mL of H_2_O. 100 μL of CAS reagent pipetted in the well of the 96 Microwell plate, then 100 μL of the tested compound solution were added at 0.125, 0.25, 0.5 and 1 mg/mL into the same well and allowed to react for 30 min. The assay was done in triplicate. EDTA was used as a positive control, H_2_O as the negative control.

### Chemistry

#### Synthesis of triethyl 2-hydroxypropane-1,2,3-tricarboxylate (TEC)

In a two-neck round bottom flask, CA (2 g, 10.4 mmol) was dissolved in EtOH (52 mL) under nitrogen atmosphere. Thionyl chloride (2.5 mL, 34.3 mmol) was added dropwise and the mixture was heated at 78 °C under reflux for 16 h. After evaporation of the solvent, the crude mixture was dissolved in a saturated solution of NaHCO_3_ and the aqueous phase was extracted with ethyl acetate three times. The collected organic layers were washed with brine and dried over Na_2_SO_4_. The residue was purified by column chromatography (hexane/ethyl acetate 6:4) to afford **TEC** as a colorless oil (2.7 g, 94%). R_*f*_ = 0.6 (hexane/ethyl acetate 1:1 + 2 drops of acetic acid, stain: PMA). HRMS (ESI) m/z calcd for C_12_H_20_O_7_Na, [M + Na]^+^: 299.1107, found: 299.1107. ^1^H NMR (400 MHz, CDCl_3_) *δ* 4.31 (q, *J* = 7.1 Hz, 2H), 4.17 (q, *J* = 7.1 Hz, 4H), 2.91 (d, *J* = 15.5 Hz, 2H), 2.80 (d, *J* = 15.6 Hz, 2H) 1.33 (t, *J* = 7.1 Hz, 3H), 1.27 (t, *J* = 7.1 Hz, 6H). ^13^C NMR (101 MHz, CDCl_3_) *δ* 173.37, 169.74, 73.20, 62.28, 60.93, 43.34, 14.07, 14.01.

#### Synthesis of (*R*)-5-ethoxy-3-(ethoxycarbonyl)-3-hydroxy-5-oxopentanoic acid (3)

In a round bottom flask, TEC (2.7 g, 9.76 mmol) was dissolved in of phosphate buffer (pH = 8) (57 mL) and *imm*-CaLB (700 mg) was added. The mixture was stirred at 40 °C for 16 h. The reaction was not complete and other 200 mg of the enzyme were added and the stirring was continued for other 60 h at room temperature. The pH of the solution was adjusted to 9 with 1 N NaOH and the aqueous phase was extracted twice with diethyl ether. The aqueous phase was acidified with concentrated HCl until pH = 3 and extracted twice with ethyl acetate. The combined organic phases were washed with brine and dried over Na_2_SO_4_. After evaporation of the solvent compound **3** (2.25 g, 93%) was obtained as a yellow oil. R_*f*_ = 0.22 (DCM/MeOH 9:1, stain: Pancaldi solution). Spectroscopic data were in agreement with already reported literature [19]. [α]_D_ =  + 3.59 (c 0.2, MeOH). HRMS (ESI) m/z calcd for C_10_H_16_O_7_Na, [M + Na]^+^: 299.0796, found: 271.0794. ^1^H NMR (400 MHz, CDCl_3_) *δ* 4.27 (q, *J* = 7.1 Hz, 2H), 4.14 (q, *J* = 7.1 Hz, 2H), 2.91 (t, *J* = 15.6 Hz, 2H), 2.81 (t, *J* = 15.9 Hz, 2H), 1.28 (t, *J* = 7.1 Hz, 3H), 1.24 (t, *J* = 7.1 Hz, 3H). ^13^C NMR (101 MHz, CDCl_3_) *δ* 174.77, 173.19, 169.80, 73.02, 62.55, 61.11, 43.19, 43.03, 14.05, 13.99, 13.95.

#### Synthesis of 5-ethoxy-3-(ethoxycarbonyl)-3-hydroxy-5-oxopentanoic acid (*rac*-3)

In a round bottom flask, to a solution of triethyl citrate (100 mg, 0.36 mmol) in EtOH/H_2_O 1:1 (1.7 mL), 0.1 N solution of NaOH (1.8 mL) was added. The mixture was stirred at rt for 5 h, then concentrated under reduced pressure. The crude was extracted twice with ethyl acetate, the resulting aqueous phase was acidified at pH = 3 and extracted three times with ethyl acetate. The combined organic phases were washed with brine and dried over Na_2_SO_4_. After evaporation of the solvent compound** 3**-*rac* (2.25 g, 93%) was obtained as a yellow oil. R_*f*_ = 0.22 (DCM/MeOH 9:1, stain: Pancaldi solution). Spectroscopic data were in agreement with already reported literature [18]. ^1^H NMR (400 MHz, DMSO-*d*_*6*_) *δ* 4.10 (q, *J* = 7.1 Hz, 2H), 4.03 (q, *J* = 7.1 Hz, 2H), 2.84 (d, *J* = 15.0 Hz, 1H), 2.79 (d, *J* = 15.5 Hz, 1H), 2.71 (d, *J* = 15.0 Hz, 1H), 2.64 (d, *J* = 15.5 Hz, 1H), 1.19 (t, *J* = 7.1 Hz, 3H), 1.16 (t, *J* = 7.1 Hz, 3H).

#### Synthesis of tetraethyl 2,2'-((butane-1,4-diylbis(azanediyl))bis(2-oxoethane-2,1-diyl))(2*R*,2'*R*)-bis(2-hydroxysuccinate) (4) and diethyl 2,2'-((3*R*,3'*R*)-butane-1,4-diylbis(3-hydroxy-2,5-dioxopyrrolidine-1,3-diyl))diacetate (5) and diethyl (*R*)-2-(2-((4-((*R*)-3-(2-ethoxy-2-oxoethyl)-3-hydroxy-2,5-dioxopyrrolidin-1-yl)butyl)amino)-2-oxoethyl)-2-hydroxysuccinate (6)

In a two-neck round bottom flask, under nitrogen atmosphere, mono acid **3** (200 mg, 0.81 mmol) was dissolved in dry DMF (6 mL). At 0 °C TBTU (305 mg, 0.95 mmol), HOBt (128 mg, 0.95 mmol) and DIPEA (0.65 mL, 3.74 mmol) were added. After 5 min stirring, putrescine (34 μL, 0.34 mmol) was added. The reaction was left for 16 h at room temperature. After evaporation of the solvent, the crude was purified by column chromatography (DCM/MeOH 98:2 → 90:10) to obtain **5** as the main product (106 mg, 69%) as a yellow oil. R_*f*_ = 0.33 (DCM/MeOH 95:5, stain: Pancaldi solution). [*α*]_D_ = –16.03 (c 0.3, MeOH). HRMS (ESI) m/z calcd for C_20_H_28_N_2_O_10_Na, [M + Na]^+^: 479.1642, found: 479.1644. ^1^H NMR (400 MHz, CD_3_OD) *δ* 4.13 (qd, *J* = 7.1, 1.8 Hz, 4H), 3.61–3.50 (m, 4H), 3.07 (d, *J* = 5.5 Hz, 2H), 3.02 (d, *J* = 4.0 Hz, 2H), 2.92 (d, *J* = 16.7 Hz, 2H), 2.66 (d, *J* = 18.3 Hz, 2H), 1.71–1.56 (m, 3H), 1.25 (t, *J* = 7.1 Hz, 6H). ^13^C NMR (101 MHz, CD_3_OD) *δ* 178.90, 175.47, 170.08, 71.77, 60.67, 41.62, 40.37, 37.65, 24.33, 13.05.

**4**: HRMS (ESI) m/z calcd for C_24_H_40_N_2_O_12_Na, [M + Na]^+^: 571.2479, found: 571.2488. ^1^H NMR (400 MHz, CD_3_OD) *δ* 4.24 (qd, *J* = 7.1, 1.6 Hz, 4H), 4.13 (q, *J* = 7.1 Hz, 4H), 3.21–3.15 (m, 4H), 2.95 (d, *J* = 15.5 Hz, 2H), 2.76 (d, *J* = 15.5 Hz, 2H), 2.75 (d, *J* = 14.6 Hz, 2H), 2.64 (d, *J* = 14.6 Hz, 2H), 1.30 (t, *J* = 7.1 Hz, 6H), 1.26 (t, *J* = 7.1 Hz, 6H). ^13^C NMR (101 MHz, CDCl_3_)* δ* 173.63, 170.19, 169.45, 73.72, 62.42, 61.05, 44.47, 43.13, 38.98, 29.70, 26.53, 14.09.

**6**: HRMS (ESI) m/z calcd for C_22_H_34_N_2_O_11_Na, [M + Na]^+^: 525.2060, found: 525.2059. ^1^H NMR (400 MHz, CD_3_OD) *δ* 4.24 (qd, *J* = 7.1, 1.7 Hz, 2H), 4.17–4.08 (m, 4H), 3.54 (t, *J* = 6.9 Hz, 2H), 3.19 (t, *J* = 6.8 Hz, 2H), 3.12–3.01 (m, 2H), 2.99–2.86 (m, 2H), 2.83–2.54 (m, 4H), 1.72–1.57 (m, 2H), 1.52–1.46 (m, 2H), 1.41–1.14 (m, 9H). ^13^C NMR (101 MHz, CDCl_3_) *δ* 178.42, 174.58, 173.57, 170.20, 170.08, 169.33, 73.60, 72.31, 62.40, 61.32, 61.03, 44.47, 43.00, 42.07, 41.03, 38.82, 38.42, 29.67, 26.42, 24.45, 14.04.

#### Synthesis of 2,2'-((3*R*,3'*R*)-butane-1,4-diylbis(3-hydroxy-2,5-dioxopyrrolidine-1,3-diyl))diacetic acid ((*R*,*R*)-glomuferrin, 1)

In a round bottom flask, to a suspension of diethyl ester **5** (29 mg, 0.07 mmol) in distilled water (7.5 mL), *imm*-CaLB (230 mg) was added. The reaction was left stirring at 240 rpm at 40 °C for 16 h. The solvent was evaporated under reduced pressure to afford pure compound **1** (26.1 mg, 96%) as a colourless oil. R_*f*_ = 0.16 (DCM/MeOH 9:1, stain: Ninhydrin solution). [*α*]_D_ =  + 1.12 (c 0.3, DMSO). HRMS (ESI) m/z calcd for C_16_H_19_N_2_O_10_, [M – H]^–^: 399.1040, found: 399.1039. ^1^H NMR (400 MHz, D_2_O) *δ* 3.59–3.42 (m, 4H), 3.10 (d, *J* = 18.6 Hz, 2H), 2.99 (d, *J* = 16.8 Hz, 2H), 2.92 (d, *J* = 16.8 Hz, 2H), 2.78 (d, *J* = 18.6 Hz, 2H), 1.60–1.40 (m, 4H). ^13^C NMR (101 MHz, D_2_O) *δ* 180.40, 177.75, 173.92, 72.22, 41.56, 41.20, 38.30, 23.98.

#### Synthesis of (2*R*,2'*R*)-2,2'-((butane-1,4-diylbis(azanediyl))bis(2-oxoethane-2,1-diyl))bis(2-hydroxysuccinic acid) ((*R*,*R*)-rhizoferrin, 2)

In a round bottom flask, compound **5** (36 mg, 0.07 mmol) was dissolved in 0.2 mL of methanol, 0.6 mL of THF and 0.2 mL of water. Then LiOH monohydrate (55.13 mg, 1.31 mmol) was added. The reaction mixture was stirred for 16 h at room temperature. Other 27.56 mg of LiOH were added and the reaction mixture was stirred for further 72 h. The solvent was evaporated and the product, dissolved in 2 mL of water, was eluted through a cation exchange resin column (Amberlyst 15^®^). Residual water was evaporated to give product **2** (29.8 mg, quantitative yield) as a colourless glass. [α]_D_ = – 2.61 (c 0.3, H_2_O). HRMS (ESI) m/z calcd for C_16_H_23_N_2_O_12_, [M–H]^–^: 435.1251, found: 435.1254. ^1^H NMR (400 MHz, D_2_O) *δ* 3.20–3.06 (m, 4H), 3.01 (d, *J* = 16.1 Hz, 2H), 2.75 (d, *J* = 16.2 Hz, 2H), 2.74 (d, *J* = 14.5 Hz, 2H), 2.62 (d, *J* = 14.5 Hz, 2H), 1.54–1.35 (m, 4H). ^13^C NMR (101 MHz, D_2_O) *δ* 176.62, 173.50, 170.94, 73.67, 44.63, 43.17, 38.88, 25.64.

#### Synthesis of 2,2'-(butane-1,4-diylbis(3-hydroxy-2,5-dioxopyrrolidine-1,3-diyl))diacetic acid (glomuferrin, *rac*-*1* and *meso*-1)

In a two-neck round bottom flask, under nitrogen atmosphere, citric acid (1 g, 5.20 mmol) was added, to a solution of putrescine (0.25 mL, 2.60 mmol) in 5 mL of xylene. The reaction mixture was stirred at 140 °C using a Dean-Stark apparatus for 24 h to remove the released water. After evaporation of the solvent the crude was purified by column chromatography (DCM/MeOH 95:5 → 80:20) to afford two major products, *rac*-**1** as a colourless oil (12%) and *meso*-**1** as a white solid (18%).

*rac*-**1**: HRMS (ESI) m/z calcd for C_16_H_19_N_2_O_10_, [M – H]^–^: 399.1040, found: 399.1035. ^1^H NMR (400 MHz, CD_3_OD) *δ* 3.53 (d, *J* = 6.4 Hz, 4H), 3.03 (d, *J* = 18.3 Hz, 2H), 3.02 (d, *J* = 16.9 Hz, 2H), 2.88 (d, *J* = 16.9 Hz, 2H), 2.65 (d, *J* = 18.2 Hz, 2H), 1.59 (t, *J* = 6.3 Hz, 4H). ^13^C NMR (101 MHz, CD_3_OD) *δ* 179.13, 175.72, 171.92, 71.73, 41.67, 40.23, 37.56, 24.21.

*meso*-**1**: HRMS (ESI) m/z calcd for C_16_H_19_N_2_O_10_, [M – H]^–^: 399.1040, found: 399.1036. ^1^H NMR (400 MHz, D_2_O) *δ* 3.66–3.53 (m, 4H), 3.15 (d, *J* = 18.6 Hz, 2H), 3.01 (d, *J* = 16.5 Hz, 2H), 2.89 (d, *J* = 16.4 Hz, 2H), 2.84 (d, *J* = 18.6 Hz, 2H), 1.65–1.58 (m, 4H). ^13^C NMR (101 MHz, D_2_O) *δ* 180.80, 177.99, 175.52, 72.54, 42.56, 41.76, 38.24, 23.98.

### Pyricularia oryzae spore germination and appressorium inhibition

*Pyricularia oryzae* PO21_05 (wild-type, *wt*) and PO21_O7 (QoI-resistent, *res*) were inoculated on a complete medium (CM) [27] and incubated in the growth chamber at 24 °C in the dark for 12–14 days. Then, 2 mL of sterile water were added to the mycelium and the formed conidia were scraped from the whole mycelium surface with the help of the sterile L-shaped spatula. The spore suspension was collected and filtered through a layer of sterile gauze into an Eppendorf tube to remove mycelium. The spore concentration was estimated using the Thoma hemeacytometer and adjusted to a concentration of 2 × 10^4^ conidia/mL.

Both glomuferrin and rhizoferrin were dissolved in water and were added to 100 μL of conidial suspension to obtain a final concentration of 5–2-0.5–0.5 mM. Conidia suspended in water were considered control. Azoxystrobin (Amistar, Syngenta) was used as a reference compound of fungal germination inhibition at concentrations 100 and 10 µM a.i. Twenty μL of the conidial suspension in three replicates were applied on a microscopic cover slide and incubated in a wet chamber for 24 h at 24 °C in the dark. The germination of 100 randomly chosen conidia for each treatment and replica was determined. The conidia were assigned into germination classes: NG = not germinated, G = germinated, and A = germinated with appressorium.

To assess the activity of the compounds in iron homeostasis, their activity was assessed in the presence or absence of Fe^3+^ ions (5 µM FeCl_3_), as previously described [5]. To the spore suspensions, FeCl_3_ (5 μM) was added at 0 h post inoculation (hpi), followed by the treatment with 2 mM glomuferrin or rhizoferrin at 4 hpi in three replicates. The conidia were incubated and assessed as described above.

### Statistical analysis

The statistical analyses were performed using R software, version 4.4.0, [28] within the RStudio interface, version 2025.9.1.401 [29]. The percentage data of the spore germination and appressorium formation were arcsine transformed and submitted to ANOVA, followed by Tukey’s post hoc test for multiple comparison (*p* = 0.05), using the TukeyC package [30].

## Supplementary Information

Additional file 1.

## Data Availability

All data supporting the findings of this study are available within the paper and its Supplementary Information. Should any raw data files be needed in another format they are available from the corresponding author upon reasonable request.
