# Supplementary material for: Facile synthesis and biological evaluation of glomuferrin and rhizoferrin as ferroptosis inhibitors in rice blast disease
Source: Nat Prod Bioprospect. 2026 May 4;16(1):60. doi: 10.1007/s13659-026-00619-x (PMC13136457; doi:10.1007/s13659-026-00619-x)

**Facile synthesis and biological evaluation of glomuferrin and rhizoferrin as ferroptosis inhibitors in rice blast disease**

Anna Fusetti, Francesca Annunziata, Michael S. Christodoulou, Andrea Pinto, Andrea Kunova, Salvatore Princiotto,* and Sabrina Dallavalle

*Department of Food, Environmental and Nutritional Sciences, University of Milan, via Celoria 2, 20133, Milan, Italy*

**Supporting information**

HPLC chromatograms of *rac*-(**1**) and *meso*-(**1**) Page S1

HPLC chromatograms of *rac*-(**3**) and (*R*)-(**3**) Page S2

^1^H- and ^13^C-NMR Page S3


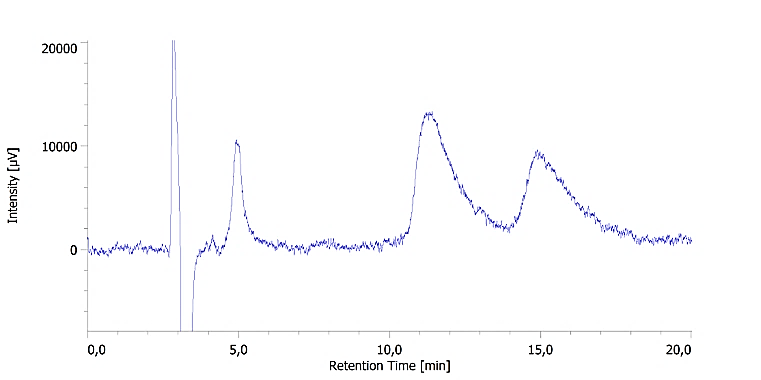

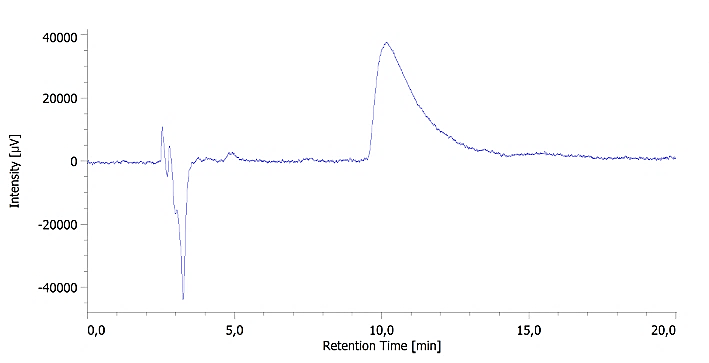


**Figure S1**. HPLC chromatogram of a) *rac*-**1** and b) *meso*-**1**. hexane:isopropanol 1:9 + 0.2% formic acid. Chiral column Kromasyl AmyCoat 5 (4.6 x 250 mm, 5 μM particle size), λ = 210 nm, Φ = 1 mL/min.


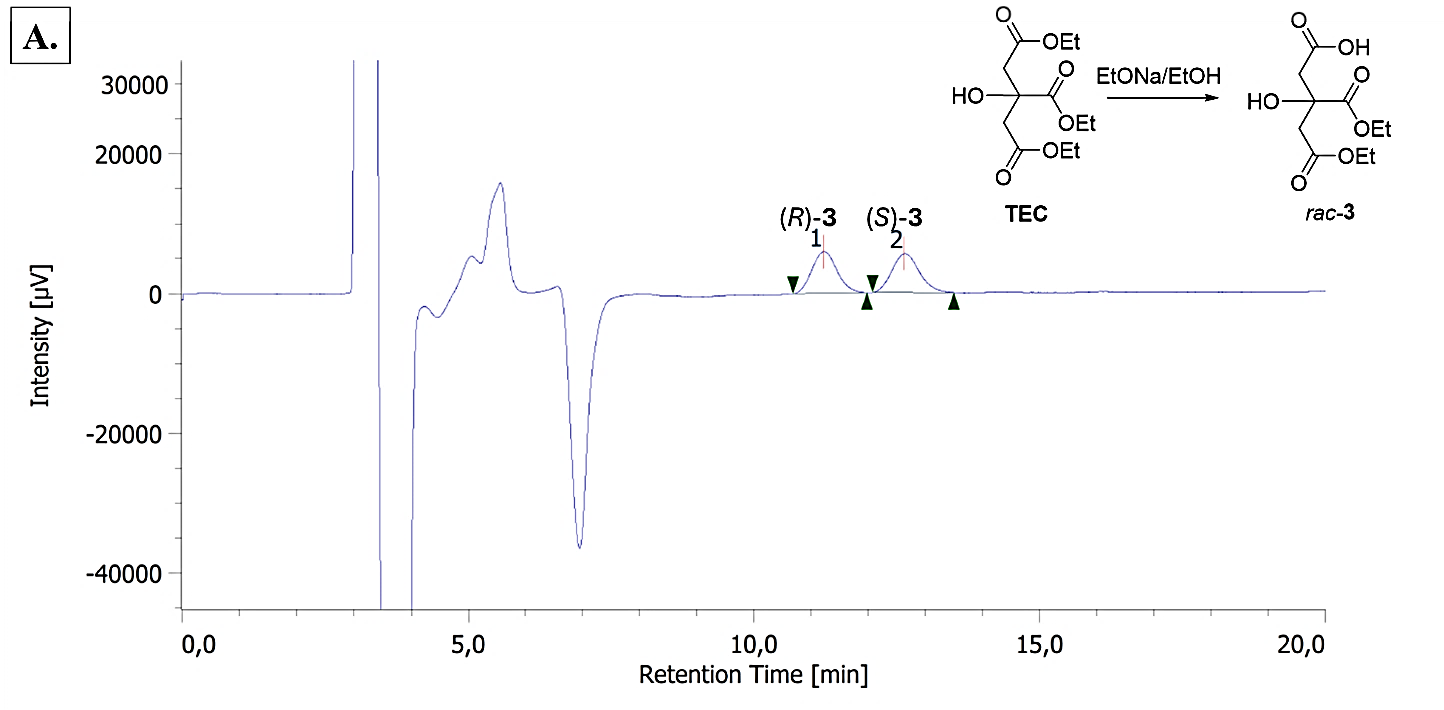


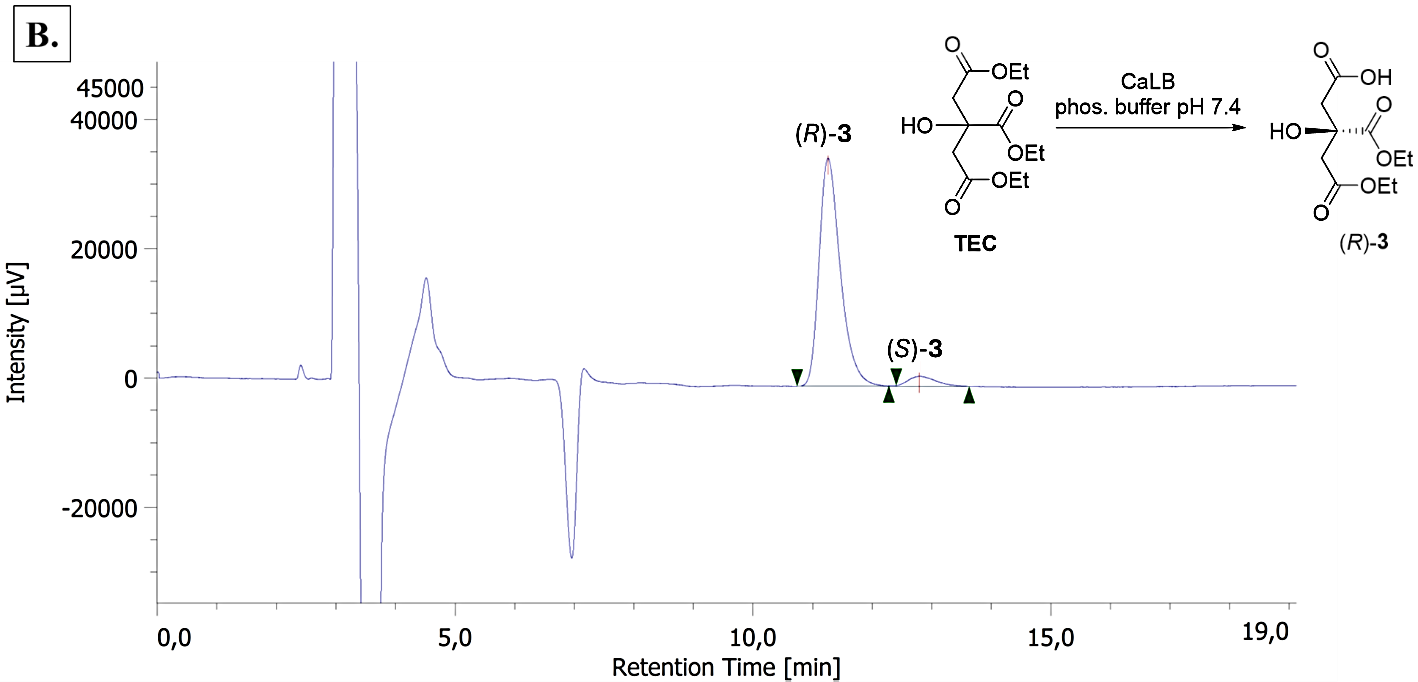


**Figure S2**. Comparison between HPLC chromatograms of *rac*-**3**, obtained by alkaline hydrolysis (A) and enantioenriched **3**, obtained by CaLB-catalyzed hydrolysis (B).


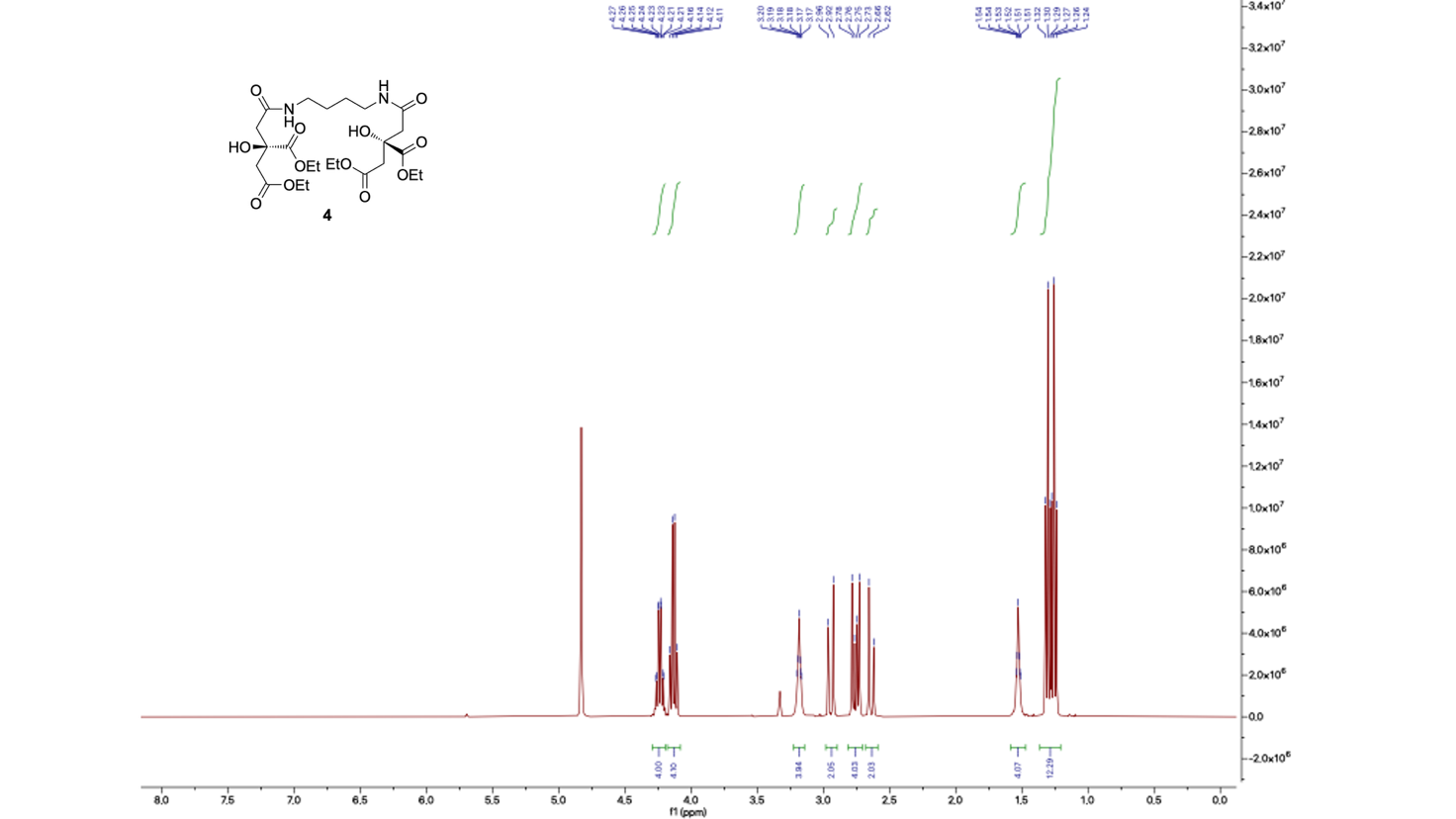


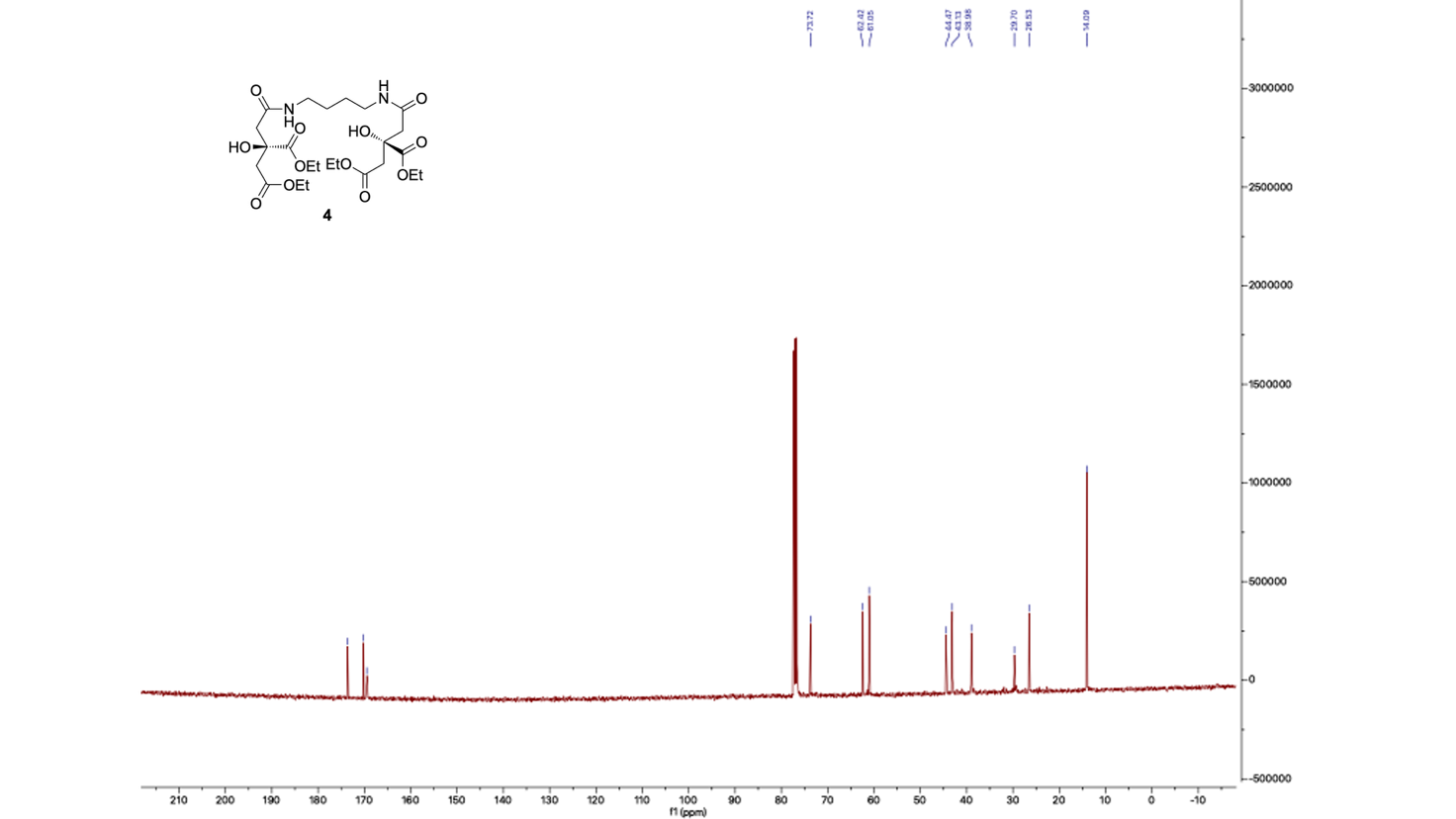


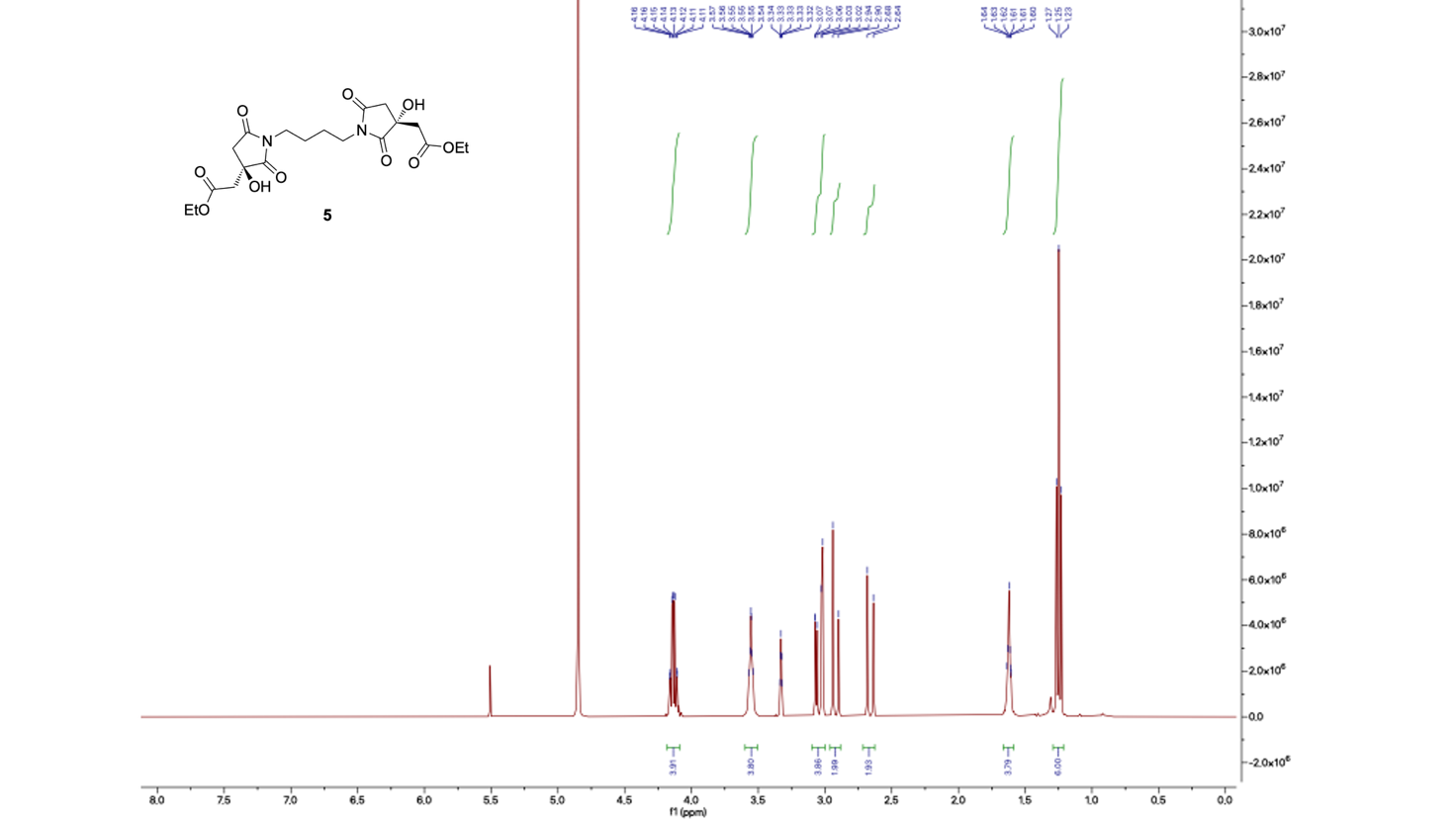


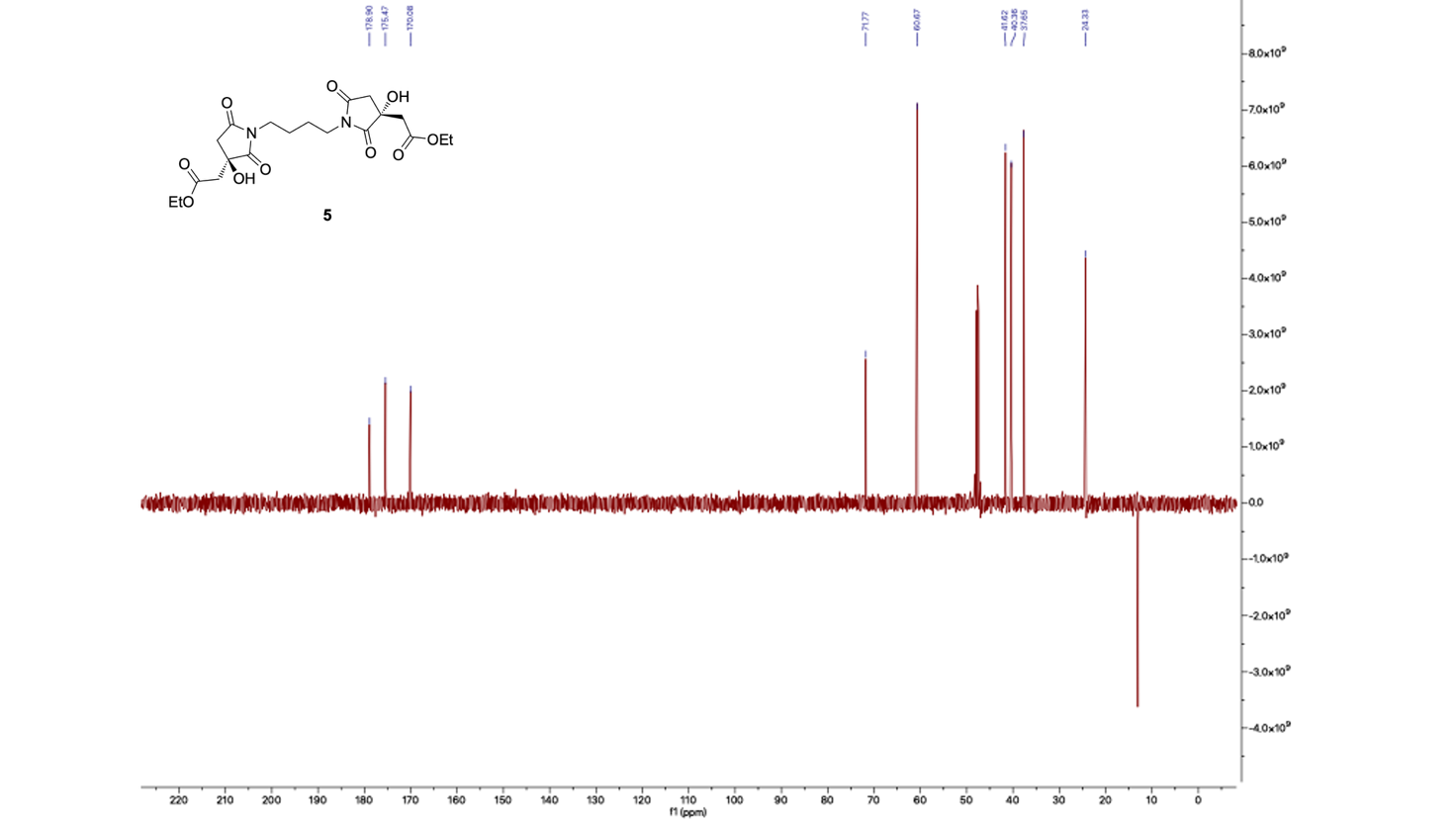


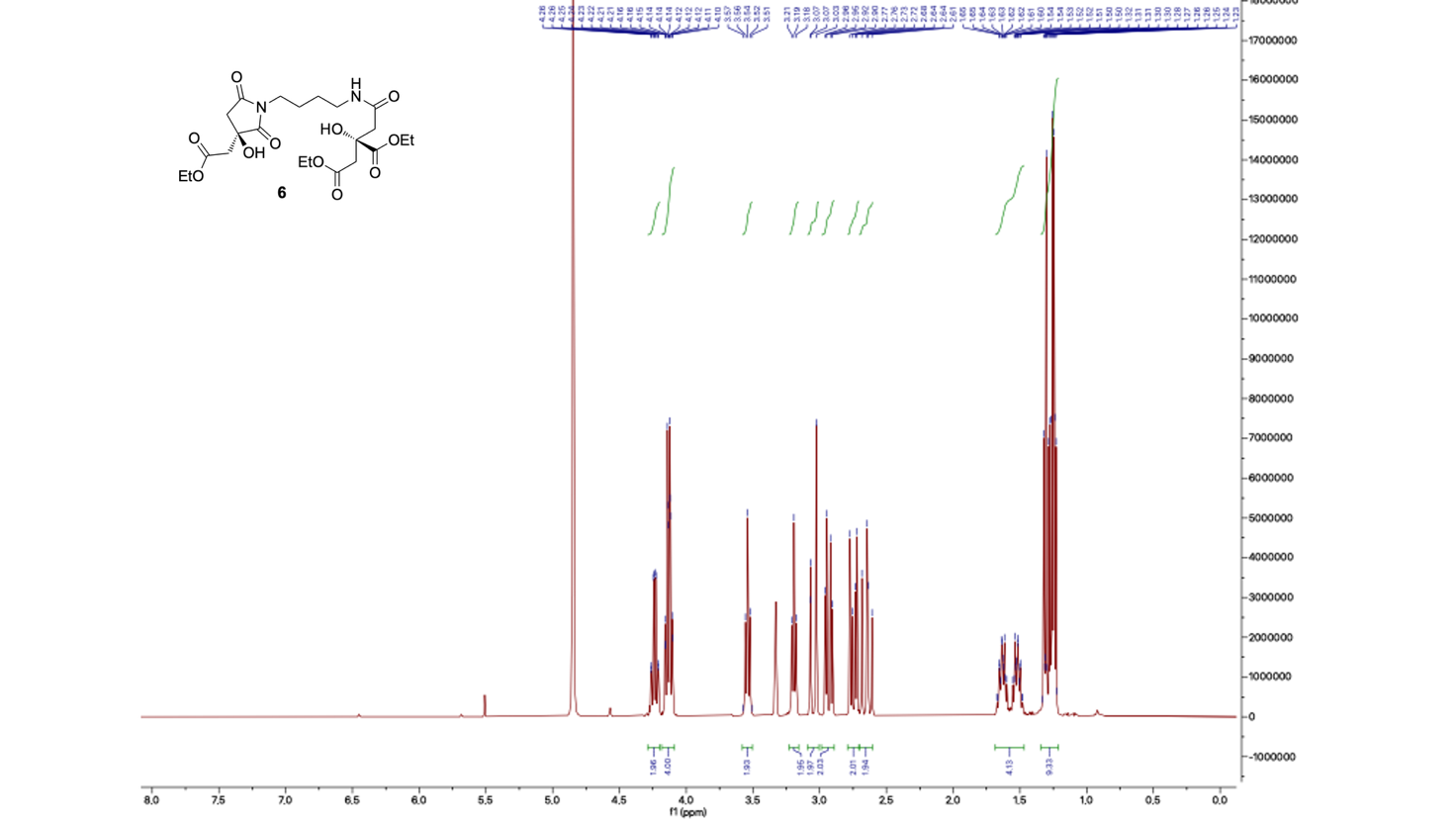


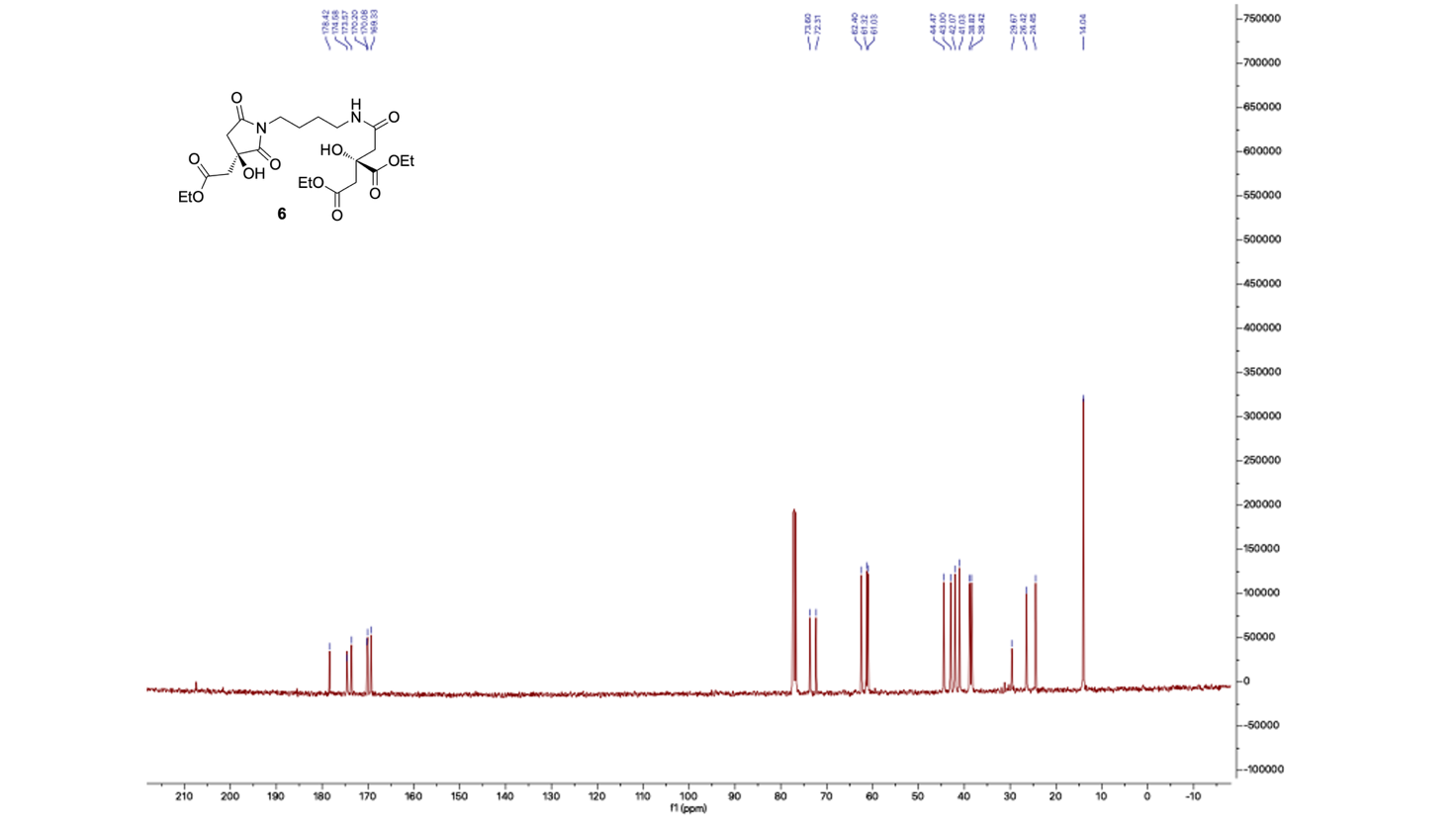


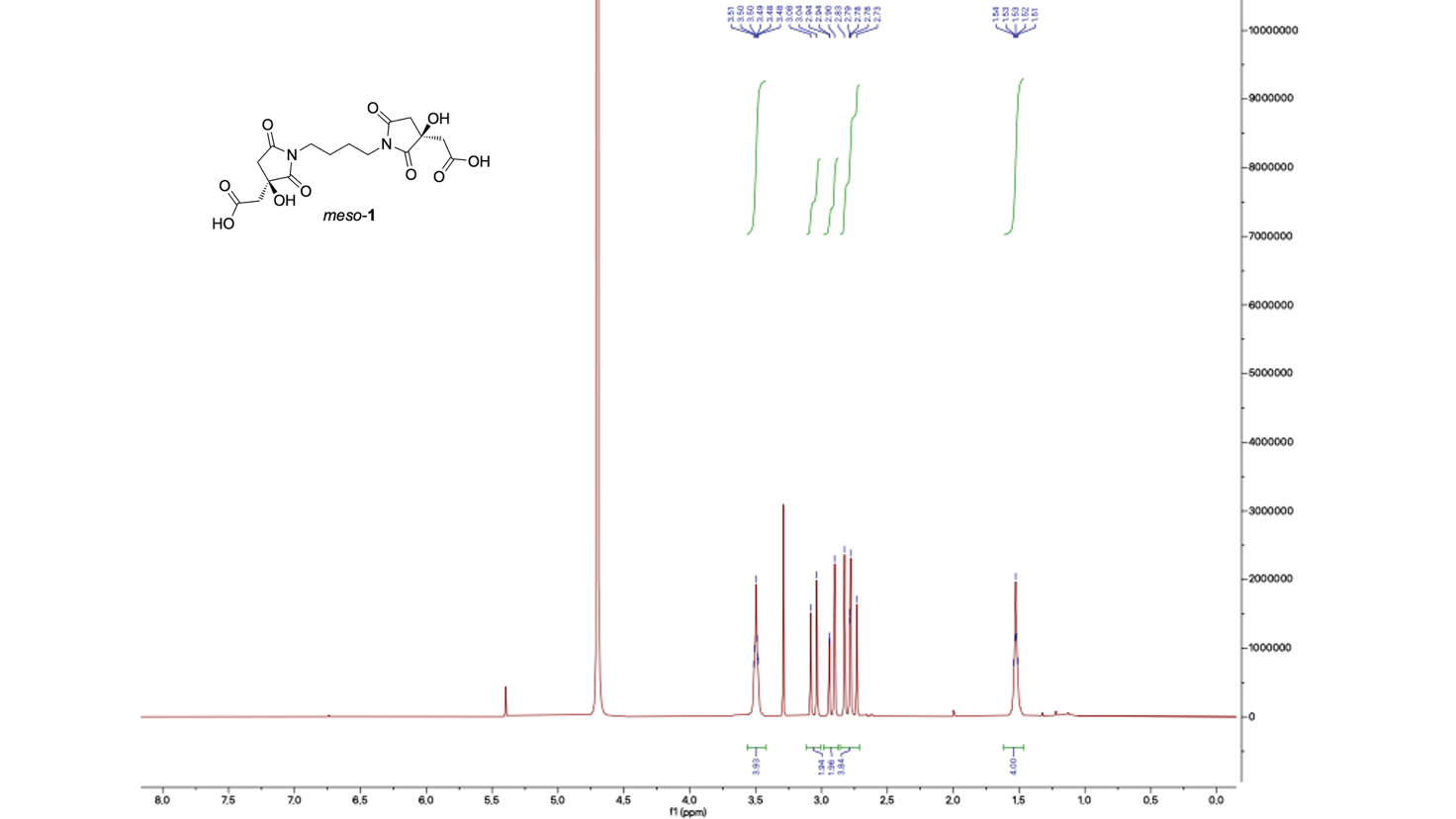


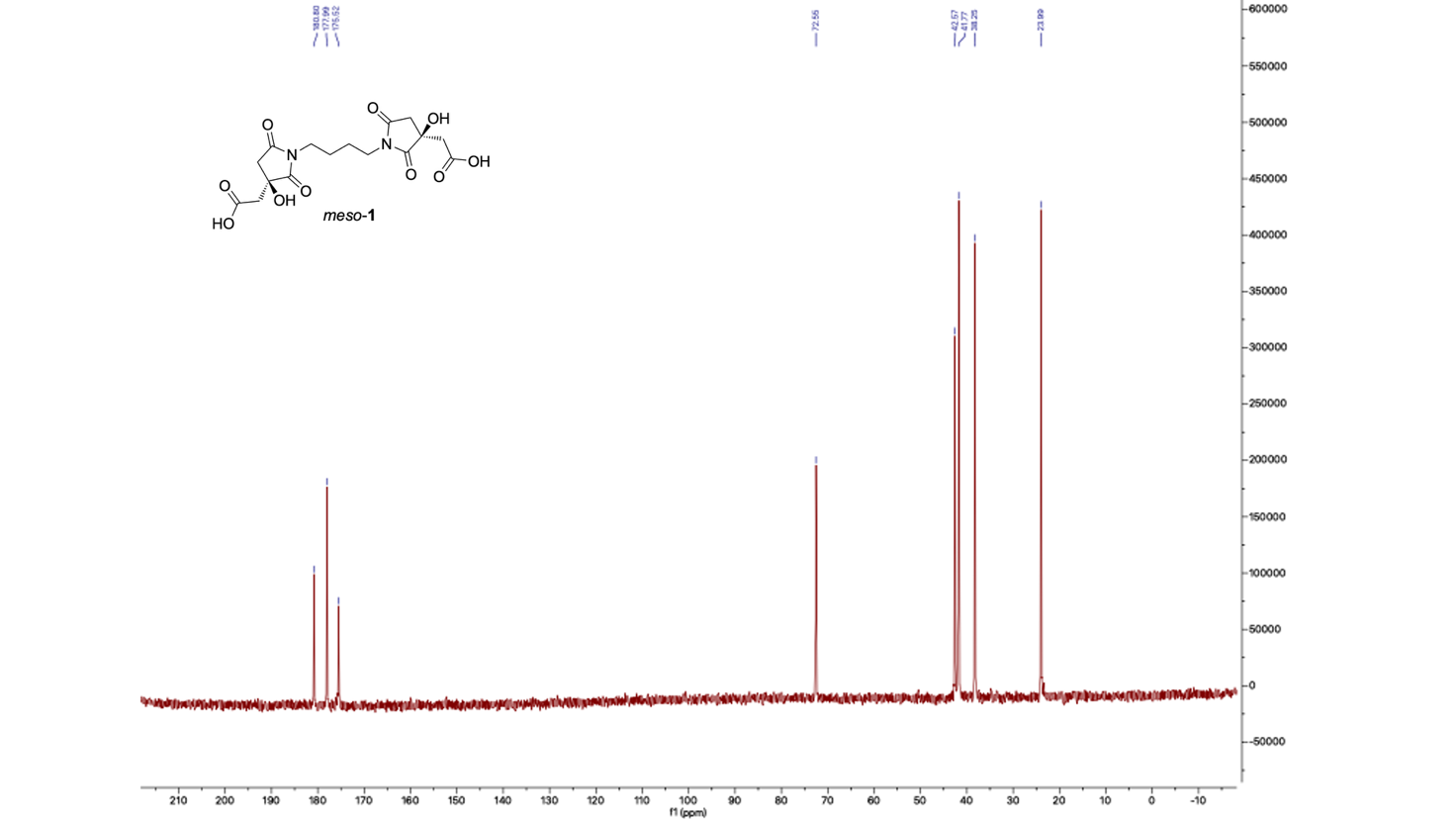

Supplement: Supplementary file 1 — Additional file 1. [file 13659_2026_619_MOESM1_ESM.docx]
